# Incentives as connectors: insights into a breastfeeding incentive intervention in a disadvantaged area of North-West England

**DOI:** 10.1186/1471-2393-12-22

**Published:** 2012-03-29

**Authors:** Gill Thomson, Fiona Dykes, Margaret A Hurley, Pat Hoddinott

**Affiliations:** 1 Maternal & Infant Nutrition and Nurture Unit, University of Central Lancashire, Preston PR1 2HE, UK; 2 School of Health, University of Central Lancashire, Preston PR1 2HE, UK; 3 Health Services Research Unit, University of Aberdeen, 3rd Floor, Health Sciences Building, Foresterhill, Aberdeen AB25 2ZD, UK

**Keywords:** Breastfeeding, Incentive, Peer support, Qualitative, Before and after cohort

## Abstract

**Background:**

Incentive or reward schemes are becoming increasingly popular to motivate healthy lifestyle behaviours. In this paper, insights from a qualitative and descriptive study to investigate the uptake, impact and meanings of a breastfeeding incentive intervention integrated into an existing peer support programme (Star Buddies) are reported. The Star Buddies service employs breastfeeding peer supporters to support women across the ante-natal, intra-partum and post-partum period.

**Methods:**

In a disadvantaged area of North West England, women initiating breastfeeding were recruited by peer supporters on the postnatal ward or soon after hospital discharge to participate in an 8 week incentive (gifts and vouchers) and breastfeeding peer supporter intervention. In-depth interviews were conducted with 26 women participants who engaged with the incentive intervention, and a focus group was held with the 4 community peer supporters who delivered the intervention. Descriptive analysis of routinely collected data for peer supporter contacts and breastfeeding outcomes before and after the incentive intervention triangulated and retrospectively provided the context for the qualitative thematic analysis.

**Results:**

A global theme emerged of 'incentives as connectors', with two sub-themes of 'facilitating connections' and 'facilitating relationships and wellbeing'. The incentives were linked to discussion themes and gift giving facilitated peer supporter access for proactive weekly home visits to support women. Regular face to face contacts enabled meaningful relationships and new connections within and between the women, families, peer supporters and care providers to be formed and sustained. Participants in the incentive scheme received more home visits and total contact time with peer supporters compared to women before the incentive intervention. Full participation levels and breastfeeding rates at 6-8 weeks were similar for women before and after the incentive intervention.

**Conclusion:**

The findings suggest that whilst the provision of incentives might not influence women's intentions or motivations to breastfeed, the connections forged provided psycho-social benefits for both programme users and peer supporters.

## Background

In the UK and internationally there has been growing interest in the use of incentives to change healthy lifestyle behaviours within an educational and public health arena [1,2]. An incentive is anything that motivates an action or behaviour and definitions differ in the published literature. Incentives may be tangible, for example gifts or awards or intangible, for example, supportive or educational relationships. Incentives may be delivered in private, for example between two individuals, or in public as in award ceremonies. Positive incentives include cash payments, grocery vouchers, T-shirts and larger rewards such as holidays, whereas negative incentives involve financial loss for non-compliance or failure to achieve an imposed target behaviour [1]. For the purpose of this study a breastfeeding incentive scheme is referred to where incentive is defined as:

*‘A thing of perceived positive value, offered in order that a desirable health outcome may be obtained, to motivate or encourage an individual to change his or her behaviour' *[2].

Whilst the terms incentive and reward are often used interchangeably, there are subtle distinctions between them. Incentives are designed to encourage individuals to adopt a specific behaviour (e.g. initiation or continuation of breastfeeding); while rewards are provided for achievement of a particular goal (e.g. breastfeeding at 6 weeks). Knowing that a single reward or a series of rewards will be given for target behaviour can also act as an incentive to both initiate and continue a particular behaviour.

There is a complex relationship between incentives and motivation, with important motives such as the desire to reciprocate and gain social approval and the intrinsic enjoyment of a behaviour interacting with tangible incentives [3]. Motivation is a human characteristic that propels us to achieve and accomplish our goals, and may operate on an intrinsic or extrinsic basis. Intrinsic motivation relates to self-determined actions that stem from the self [4]; such as the individual's internal desires to perform a particular task as it provides pleasure or enables skill development. Intrinsic motivation has been explained by psychologists such as Heider [5] through his work on attribution theory, Bandura's [6] self-efficacy theory and within the theory of planned behaviour [7]. These theories purport that individuals who are intrinsically motivated are more likely to attribute success to internal factors as opposed to external influences. Extrinsic motivation, on the other hand, relates to factors that are external to the individual, e.g. financial, to please others or due to threats of punishment [4]. Extrinsic motivators can therefore motivate people to perform certain tasks rather than purely for the pleasure of, or desire for attainment. Incentives that invoke intrinsic motivation are associated with more sustainable behavioural changes [8]. However, whilst extrinsic motivators may decrease intrinsic motivation [8], if the reward is internalised as a sign of competence, as opposed to a bribe [9] or is considered satisfying [10] intrinsic motivation may be maintained. Deci & Ryan thereby argue that extrinsically motivated rewards that facilitate choice, address emotions and provide opportunities for self-direction can increase an individual's sense of autonomy and, in turn, their intrinsic motivation [11].

Most intervention studies have investigated financial incentives [12], and from an economic theory perspective they are considered to improve the value of the target behaviour [13] or remove the barriers to a more healthy lifestyle [1]. From the existing literature, incentives appear more effective if they target a one-off behaviour, such as attending a clinic appointment [2,13]. However, with more complex behaviours such as encouraging physical activity, weight management and smoking cessation the results are generally inconclusive [12]. Furthermore there is evidence to suggest that changes in target behaviour are not sustained once the incentives have been withdrawn [1,13]. Incentives have been criticised as potentially coercive, and could even encourage unhealthy behaviours or game playing to ensure eligibility [2].

Whilst systematic reviews of breastfeeding incentives were not located, a number of studies have explored the impact of incentives on breastfeeding initiation and duration. In 1972 in the U.S.A. a Special Supplemental Food Program for Women, Infants and Children (WIC) commenced to provide support to low income pregnant and lactating women and children up to the age of 5. Since 1975, the WIC has incorporated incentives to promote breastfeeding. In the WIC intervention studies [14-19] incentives are used a) to increase participation in interventions; b) to reward breastfeeding behaviour and c) as a motivator to remain in the research trial. For example, in a randomised controlled trial of 68 WIC pregnant women, breastfeeding rates increased at hospital discharge, 6 weeks and 3 months for those receiving incentives [15]. Incentives were provided at the points of feeding outcome measurement and included a gift bag, breast-pump, $25 -$50 gift certificates, and partners received tickets for a local football game. Gifts of significantly higher monetary value were provided to those who were exclusively breastfeeding. Overall, however, the results of these trials are inconclusive, several include small samples, have methodological weaknesses and it is difficult to identify how effect sizes relate to the incentive or the education/support components.

Breastfeeding incentive intervention studies have focused on quantitative outcomes and do not explain how these incentives can mediate or moderate women's breastfeeding experiences and behaviour. Whilst some studies report that participants value a gift certificate [14]; a cross-sectional survey of 130 WIC participants in Louisiana reported that incentives did not encourage women to breastfeed [20]. There is therefore a need for further research into what incentive characteristics (type, quantity, timing and delivery) might improve breastfeeding outcomes and how they operate and interact with the intrinsic or extrinsic motivations of women to breastfeed.

In this paper findings are reported from an incentive intervention delivered within an existing breastfeeding peer support programme. The primary prospective aim was to conduct a qualitative study to explore the meanings attributed to receiving and giving incentives from the perspectives of women and peer supporters. A secondary aim was to retrospectively describe the participation in the peer support programme, the contact time with peer supporters and the breastfeeding outcomes at 6-8 weeks using routinely collected data before and after the incentive intervention.

## Methods

### Setting

The peer support programme operates in a Primary Care Trust (PCT) in the North West Strategic Health Authority (NWSHA) in England with a predominantly white ethnic background (98%) population of circa 142,000 and high deprivation indices [21]. The area has one maternity hospital with approximately 1,600 births per year and breastfeeding rates at 6-8 weeks are routinely collected by the Child Health Team. The rates of any breastfeeding (any breast milk given within the previous 24 hours) at 6-8 weeks have shown an increase from 19.2% in quarter 1 of 2008-09 (April-June, 2008) to 22.6% in the first quarter (April - June, 2010) of 2010-11. However this is low compared to the UK in 2005 where 48% were giving some breast milk at 6-8 weeks [22]. The breastfeeding peer support programme (Star Buddies) is provided by The Breastfeeding Network (BfN) [23,24]. It provides peer support in pregnancy through breastfeeding workshops, face-to-face on the hospital post-natal wards and up to 8 weeks after birth, by telephone, text messaging or face to face at home or community locations. An overview of the Star Buddies service is provided below:

The star buddies peer support programme

In 2009 the local PCT commissioned The Breastfeeding Network (BfN), a registered charity, to offer an extra tier of breastfeeding peer support to mothers before and after birth with the aim of increasing breastfeeding initiation rates and prevalence of breastfeeding at 6-8 weeks. The name Star Buddies came via suggestions across the BfN organisation and from a local publicity campaign designed to encourage young mothers to breastfeed (Be A Star campaign [25]).

This programme comprises 9 paid peer supporters and unpaid volunteer local breastfeeding mothers. Two of the peer supporters coordinate the service, 3 provide breastfeeding peer support during the antenatal/intra-partum period and 4 provide post-natal community based support. All supporters attend the accredited 'helpers' course, delivered over a 6 or 12 week period and the majority have attained 'supporter' status which comprises a 12 month training course.

The two Star Buddies coordinators (antenatal/hospital and community) hold weekly meetings which include case reviews and peer supporters researching specific topics for group discussion. All the Star Buddies are encouraged to contact the coordinators as and when issues arise within daily practice. Within the BfN organisation, all breastfeeding helpers and supporters have a named supervisor and have to attend an agreed number of supervision sessions over a 6/12 month period that comprise reflection on practice, on-going learning and adherence to policies and procedures.

Women can self-refer to the peer support programme or be referred by health or community professionals. Peer supporters aim to contact women who have enrolled onto the programme within 48 hours after hospital discharge and are offered up to 8 weeks of breastfeeding support provided through text messaging, telephone calls, home visits and breastfeeding support groups at community locations. If a woman ceases to breastfeed, the peer support discontinues and women can choose to opt in or out at any time within the 8 weeks. The post-natal service was informed by guidance produced by the National Institute for Health and Clinical Excellence [26].

### Design

The PCT and BfN commissioned a prospective qualitative evaluation of the incentive intervention (described in the following section) to investigate the barriers and facilitators to incentive uptake, their impact, and the meanings attributed to them from the perspectives of women recipients and the peer supporters delivering them. To triangulate the qualitative findings and provide contextual data, the research team then retrospectively analysed electronic data routinely collected by the PCT and peer supporters before and after the incentive intervention.

### The incentive intervention

In November 2010, the NWSHA provided approximately £15,000 to 4 maternity health trusts with the lowest breastfeeding duration rates to run a breastfeeding incentive intervention. No specific guidance was provided from the NWSHA in terms of how the incentive programme should be operationalized; rather that the intervention would reflect local needs and available resources. The aim of the incentive intervention was to improve any breastfeeding rates at 6-8 weeks by 5% at quarter 4 (January-March, 2011) compared to quarter 1 (April-June, 2010) figures.

The incentive intervention was integrated into the postnatal community part of the Star Buddies programme and ran over a period of four and a half months (November-March, 2011). From hospital discharge, peer supporters aimed to arrange a weekly home visit or meeting for 8 weeks to deliver 8 different incentives with a monetary value of £71.99 (Table 1) per woman and provide breastfeeding information and support. The incentives were referred to as gifts and were selected through consultation with peer supporters and breastfeeding women. The gifts were delivered in the same order, and were specifically chosen to facilitate targeted discussions about specific breastfeeding issues (Table 1).

**Table 1:** Details of gifts, order of receipt and rationale

Details of gifts and order of receipt	Rationale for the gift and associated discussion
• Congratulations gift - a picture frame (week 1)	To celebrate the birth of the child, and prompt discussion of how thinking/about/looking at baby can stimulate enhance breast-milk production.

• Selection of healthy treats (graze box) (week 2)	To promote a discussion on healthy eating, and the importance of a healthy lifestyle during breastfeeding
• Swimming voucher (week 6)	

• Mum's pamper gift set (week 3)	To encourage women to take time out for themselves, to relax and re-charge their energy levels for successful breastfeeding
• Choice of glossy magazine (week 4)	
• Pamper session (week 8)	
• Voucher for quality ready-made family meal deal (week 7)	

• Hot drink/cake from department store (week 5)	To initiate discussions on breastfeeding outside the home, any barriers or concerns and to promote a local Breastfeeding Friendly Business Campaign which provides a sticker to indicate that breastfeeding women are welcome

### Recruitment and participants

In total, 141 mothers who had initiated breastfeeding and signed up to the community peer support programme between 16^th ^November, 2010 to 3^rd ^February, 2011 were invited to take part in the incentive intervention. Five women refused the incentives but signed up for and received the postnatal peer support. A further 42 women were un-contactable and/or changed their minds about breastfeeding in the early post-natal period. Overall, 94 women either partially (completed some but not all of the 8 weeks of support) or fully (completed full 8 week programme of support) engaged with the incentive intervention.

**Figure 1:**
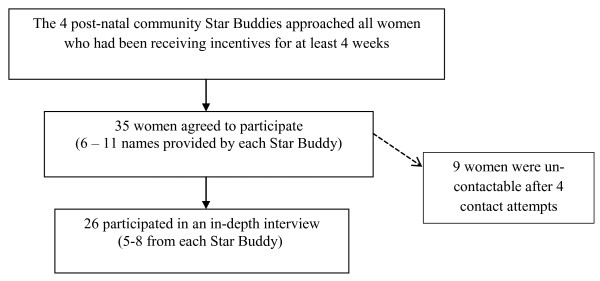
**Recruitment strategy for women receiving incentives between January - March, 2011**.

Between January to March, 2011 the community peer supporters delivering the intervention were asked to identify and verbally invite all women receiving the incentives to take part in qualitative interviews, to try and minimise response bias. The names and contact details of 35 consenting women were forwarded to GT; with each peer supporter recruiting between 6-11 women. As the aim was to elicit views of women who had received some or all of the incentives, women were only approached to participate after 4 weeks of the incentive intervention. Furthermore, whilst none of the women refused to participate, only 4 attempts were made to contact the women. An overview of the recruitment and selection process is presented in Figure 1:

All the 4 post-natal community peer supporters delivering the incentive intervention were approached and consented to participate in a focus group discussion.

### Qualitative data collection

In-depth qualitative interviews were conducted with women in receipt of the incentive peer support intervention. A semi-structured interview and a focus group schedule were devised and refined through consultation with the funders, programme providers and the wider incentive literature. The schedules were designed to explore the participants' attitudes, experiences and perceptions of the intervention, and whether the gifts had influenced their breastfeeding experiences. Where possible, interviews were organised after women had completed the 8 week peer support programme as they may have felt restricted in raising any negative appraisals whilst still in receipt of support.

In total, 26 women took part in an in-depth interview, either face to face at the women's homes (n = 9) or via the telephone (n = 17). Interviews were undertaken by three members of the research team under the direction of the project lead (GT). Multiple interviewers can add differing perspectives to an evaluation and can strengthen both the data collected, minimise the interpretive bias that can occur with a single interviewer and thus strengthen the analysis. The aim of data collection was to encourage in-depth exploration of the women's attitudes, perceptions and experiences of the incentive intervention.

The women interviewed were aged between 21 and 42 years of age, 14 (53.9%) had 1 child, 7 (26.9%) had 2 children, 4 (15.4%) had 3 and 1 (3.8%) had 5. One of the women was of Asian origin and the remaining women were White-British. Twenty-four of the women had successfully completed the 8 weeks community/incentive Star Buddies programme and two women were still in receipt of the programme. At the time of the interview, infants were aged between 6 and 16 weeks. Sixteen of the women were exclusively breastfeeding; 2 were bottle-feeding and the remaining 6 were mixed feeding, with two reporting infrequent use of formula milk. The interviews took between 30-70 minutes to complete.

A focus group discussion lasting 80 minutes was conducted with the 4 breastfeeding peer supporters who delivered the incentives and who had been working as Star Buddies for 18-22 months. This method was chosen as it concerned a non-sensitive topic area and would enable a richer exploration of their attitudes and experiences. Care was taken during the focus group to ensure that each of the peer supporters participated in the discussions, and for their views and attitudes to be expressed. The focus group was facilitated by GT.

### Qualitative data analysis

All qualitative interviews and the focus group were digitally recorded with informed consent and transcribed in full. Qualitative data analysis was undertaken through an iterative process of reading, analysing and writing to form basic, organizing and global themes using thematic networks analysis [27]. Data analysis was supported by the MAXQDA qualitative software package. Initial data analysis was undertaken by GT who had in-depth knowledge of the peer support programme from an earlier evaluation [24]. Initial readings of the transcripts involved the identification of emergent themes and a mapping framework was constructed. This framework was subsequently utilised across all the transcripts with amendments made as appropriate, with deviant cases identified and acknowledged. Trustworthiness of the findings was undertaken through the interpretations being regularly discussed and shared with all members of the evaluation team and the programme providers through regular attendance at the Star Buddies Steering Group. The key themes were also forwarded to all the participants with requests for feedback to be returned within a one month period. Two women and all the peer supporters responded to highlight their full agreement with the key themes identified.

### Quantitative data collection and analysis

Quantitative data collection and analysis took place after the qualitative data analysis was complete. Since April 2008, the UK Department of Health (DH) have requested all PCTs to submit routinely collected and reported breastfeeding duration rates at 6-8 weeks quarterly for infants who are: a) totally breastfed; b) providing 'some' breast milk (i.e. also receiving formula or other liquids) and c) not being breastfed at all. Health visitors collect the data at the universal 6-8 week child health development assessment.

Peer supporters record data for each new woman at registration with the programme, including maternal age, parity, types of peer support provided, total contact time spent with women, programme completion rates and feeding outcomes (exclusive breastfeeding and any breastfeeding) at 6-8 weeks. Since July 2010 data has been entered into Excel spread sheets by the programme administrator and these were accessed for peer support prior to (1^st ^July- 15^th ^November, 2010) and after (16th November - 31^st ^March, 2011) the incentive intervention commenced. Descriptive analysis was conducted using SPSS v. 17. The evaluation team extracted data on all the women who registered with the peer support programme, including those who fully or partially participated and women who became un-contactable and/or changed their minds about breastfeeding in the early post-natal period. All women completing the full breastfeeding peer support programme were giving their infant some breast-milk (exclusively or mixed feeding) at 6-8 weeks as this is a requirement for receiving the peer support. Descriptive analysis was undertaken to triangulate the qualitative findings and to provide important contextual information for the commissioned qualitative thematic analysis.

### Ethics

The project was reviewed by the National Research Ethics Committee and received ethics approval by the Faculty of Health Research Committee at the lead author's university, and the Research & Design Unit at the sponsoring NHS Trust. Ethical principles to ensure informed consent prior to interview, autonomy and confidentiality were adhered to in the evaluation. Pseudonyms have been used to protect the anonymity of the women.

## Results

### Outcomes of the peer support and incentive intervention

From the routinely collected infant feeding data for all women giving birth in the study area (Table 2), the 'any' breastfeeding rates at 6-8 weeks were 22.6% (n = 93) at quarter 1 (April-June, 2010) and increased to 29.9% (n = 123) by quarter 4 (January-March, 2011). This represents an increase of 7.3%, thereby exceeding the 5% target set by the NWSHA for the incentive intervention; with the quarter 4 duration rates being the highest since routine monitoring of this data was introduced.

**Table 2:** Routinely collected quarterly infant feeding outcomes collected by health visitors at the 6-8 week child development assessment

Feeding method at 6-8 weeks	Quarter period and number (%) babies					
	**January - March, 2010**	**April - June, 2010**	**July - September, 2010**	**October - December, 2010**	January - March, 2011*	**April - June, 2011**

**Breast milk only (exclusive breastfeeding)**	82 (18.3%)	76 (18.5%)	85 (19.4%)	73 (18.7%)	99 (24.1%)	78 (22.0%)

**Mixed breast and formula milk**	30 (6.7%)	17 (4.1%)	29 (6.6%)	21 (5.4%)	24 (5.8%)	21 (5.9%)

**Some breast milk (exclusive and mixed)**	112 (25%)	93 (22.6%)	114 (26%)	94 (24.1%)	123 (29.9%)	99 (27.9%)

**Formula milk only**	322 (71.7%)	316 (76.9%)	304 (69.2%)	277 (71.0%)	288 (70.0%)	247 (69.6%)

**Unknown**	15 (3.3%)	2 (0.5%)	21 (4.8%)	19 (4.9%)	0	9 (2.5%)

* The highlighted column in the table reflects the period when the incentive intervention was running

Between July 2010 and March, 2011 a total of 408 women registered with the peer support programme, 272 before and 136 after the incentive intervention started (Table 3).

**Table 3:** Comparison of participation, breastfeeding outcomes at 6-8 weeks and contacts with peer supporters before and after the incentive intervention

	Before Intervention: Registration for the peer support programme 1^st ^July - 15^th ^November 2010	After Intervention: Registration for the peer support and incentive intervention 16^th ^November 2010^1 ^- 3^rd ^February, 2011
Registered as interested in participating	272	136

**Fully (8 weeks) or partially (< 8 weeks) participatingn (%)**	**172 (63.2%)**	**94 (69.1%)**
		

Age (years) mean (SD)	28.8 (6.0)^a^	29.4 (5.3)^b^

Some breastfeeding (exclusive or mixed) at 6-8 weeks (%)	119 (69.2%)	57 (60.6%)

Number of home visits mean (SD)	0.9 (1.1)	3.3 (2.8)

Total contact time (minutes) mean (SD)	145.8 (165.6)	225.3 (161.6)

**Completed full 8 week programme of support and therefore providing some breast milk at 6-8 weeks n (%)**	**108 (39.7%)**	**53 (38.9%)**

Age (years) mean (SD)	29.8 (5.6)^a^	30.2 (5.1)^c^

Exclusive breastfeeding at 6-8 weeks n (%)	74 (68.5%)	40 (75.5%)

Number of home visits mean (SD)	1.0 (1.2)	5.0 (2.4)

Total contact time (minutes) mean (SD)	169.8 (193.0)	313.2 (151.2)

Missing data: ^a ^3 cases; ^b ^4 cases; ^c ^1 case

^1 ^The five women who were eligible to receive the intervention, but declined to participate have been excluded from the data set.

With regard to the women who were registered for the peer support service before the incentive intervention, 172 of 272 (63.2%) women participated either fully or partially; with 108 (39.7%) completing the full 8 week support programme.

From the 136 eligible women who were registered after the incentive intervention started, 94 of 136 (69.1%) participated (fully or partially); with 53 (38.9%) completing the full 8 week programme. Maternal age before and after the incentives intervention was similar for participants.

Before the incentive intervention, 119 of the 172 participants (69.2%) were giving their baby some breast milk at 6-8 weeks compared to 57 of the 94 participants (60.6%) of those who received the incentives. For women who completed the full 8 week peer support programme, 74 women (68.5%) were exclusively breastfeeding before the incentive intervention compared to 40 women (75.5%) who received the incentives. Women participating in the incentive intervention received a mean of 3.3 home visits compared to 0.9 before the incentive intervention. Similarly, the mean total contact time with peer supporters was considerably higher for the incentive intervention (225 minutes) compared to the peer support programme alone (145 minutes).

### Qualitative data

The global theme to emerge from the qualitative data was 'incentives as connectors'. Two organising themes are presented, 'facilitating connections' and 'facilitating relationships and wellbeing'. The themes and associated sub-themes are detailed in Table 4. These themes explore how the incentives and opportunities to develop connected relationships provided tangible and intangible benefits for women and peer supporters.

**Table 4:** Global, organising and basic themes

Global theme	Organising themes	Basic themes
**Incentives as Connectors**	*Facilitating Connections*	Encouraging access
		Connecting to self and others
		Relating to outside world
	
	*Facilitating Relationships and Wellbeing*	Being on the journey together
		Encouraging sensitive dialogues and opportunities for support
		Being rewarded

#### Facilitating connections

This theme explores how the provision of, and quality of the gifts were 'encouraging access' between the peer supporters and mothers; how the gifts enabled 'connections to self and others' and how the gifts facilitated mothers 'relating to the outside world'. 

#### **Encouraging access**

The Star Buddies reported how gifts enabled them to gain a '*foot in the door' *into the women's homes and lives. Whilst women in receipt of the 'usual' programme were generally happy to receive on-going breastfeeding support via text or phone, face-to-face contact was often limited until a specific breastfeeding problem emerged. However, delivering the gifts facilitated repeated face to face contact with women:

*‘I suppose the thing about the gift scheme is you're signed up to see them every week, to have contact with them every week...' *(Mary).

The actual quality of the gifts was identified as enabling regular access to women. Highly positive comments were made regarding the gift variety, their appropriateness, the targeted discussions they stimulated, and the thought and care that had gone into their selection:

*‘It was a really, really nice touch I thought and the gift themselves were very, very well thought out, in the way that they gave like the healthy snacks and the magazine, which is great to have when you're breastfeeding. Every gift that I received was really appropriate and I've enjoyed every one, it's been really good' *(Rose).

The quality of the gifts meant that the Star Buddies were more willing to impart the presents; *'we were happy to hand them over'*. Pleasure was obtained through accounts and observations of the gifts being utilised, and from women's comments:

*‘...What was lovely was seeing the picture frames getting the picture in and being on the mantle*.....

*...'I had a couple go swimming and they've been really pleased' *(Peer Supporters).

The gift-giving enabled peer supporters to provide regular proactive weekly support to mothers identified as difficult to access, e.g. multi-parous, younger and vulnerable mothers:

*‘I'm glad I ended up *[seeing the Star Buddies] *because I don't have loads of people come to my house, I'm quite a private person....... but overall, if I did have questions, she was there and she'd reassure me, so I'd feel reassured rather than panicking and thinking, oh I don't know what's what' *(Nicky).

#### Connecting to self and others

A recurrent message to emerge across all the narratives was how the gifts were tailored towards them and the families. Women repeatedly cited how the gifts reminded them of their individuality. As mothers can lose their identity as they adjust to the parenting role, these gifts were considered to re-connect them to their sense of self:

*‘I think it reminds you of you being an individual. I've been constantly like X (son) on the breast and sorting the kids out and she came with a gift and it's like, oh yes, this is for me.....who am I again? It reminds you that you need to look after yourself as well sort of thing' *(Nicky).

Peer supporters and the mothers frequently reflected how the birth of a baby can leave mothers feeling overwhelmed and preoccupied in adapting to their new role. These gifts therefore often provided women with the feeling of being cared for and the need for self-care:

*‘It was nice we got, I think on the second or the third week, I can't remember, they give you .....I think it was a pamper set, like body scrub and bath stuff, that was really nice, because I felt like I'm not paying any attention to myself since she was born, so that was nice' *(Claire)

Furthermore, the nature and quality of the gifts, and associated discussions with peer supporters could motivate women to initiate quality time with their partner, families and babies:

*‘Even a simple gift like a cup of coffee and a voucher, it seems like giving us, me and my husband, time to spend outside' *(Nadia).

From a peer supporter perspective, the increased opportunities for home visits enabled identification of worries or concerns, which subsequently brought the Star Buddies into closer contact with other health professionals to try and resolve them. These occasions helped to raise awareness of the Star Buddies programme, promoted extended contact between the peer supporters and health professionals and facilitated the development of more collaborative relationships:

*‘I've had a lot more contact phoning midwives and health visitors to say mum's worried about this and she's asked me to speak to you and.....they've also contacted us. We have had more contact with health visitors and things' *(Peer Supporters).

#### Relating to the outside world

Women's regular contact with the Star Buddies provided them with a *'life-line' *to the outside world. Women looked forward to the visits which helped to safeguard against maternal isolation:

*‘Because when you're sitting in your house on your own and your other half is at work, and it's just you day after day after day at home with your child, you begin to feel very isolated and you begin to feel very on your own. And having her coming every Friday, you know, it's a colossal difference' *(Erica).

The gift(s) and associated discussions encouraged women to breastfeed outside the home environment and promoted access to wider support networks. Peer supporters reported that the number of women accessing the breastfeeding groups increased over the incentive programme. Moreover, as some of the women preferred contact at community locations, these opportunities encouraged women to gain access to additional social occasions and activities:

*‘Because we've got them into lots of groups, they all come to baby groups, baby massage, baby yoga. Because they're here and they know us all and then they join the centre and do other stuff, it's like a community almost' *(Peer Supporters).

#### Facilitating relationships and wellbeing

This theme reports on how the provision of gifts and repeated contacts with women facilitated meaningful relationships through 'being on the journey together'; 'encouraging sensitive dialogues and opportunities for support' and how the incentive intervention provided rewards for women as well as the peer supporters.

#### Being on the journey together

The peer supporters considered that regular face-to-face access to women, couched within the provision of gifts enabled a more meaningful and connected relationship to be forged:

*‘We had real relationships, rather than the actual giving of the gift, though that was nice, you had a way to get in the door and once you're in the door you could build on all sorts of things' *(Peer Supporters).

Whilst 'friendships' being forged between peer supporters and mothers was evident, the incentive scheme created continuity and a situation in which the peer supporters '*were with them along their journey'*. Repeated contacts enabled the supporters to monitor the on-going health of the woman and infant and to be cognisant of women's and families values and beliefs:

*‘With X *(Star Buddy), *knowing that he was a placid baby and then suddenly changing into this crazy, screaming banshee that he was. And the fact that she rang the health visitor and said, look, no he's not himself, you know, he's being different, I think that really helped' *(Shona).

Previously Star Buddies felt pressured to impart as much information whenever opportunities for contact arose. The weekly home visits provided sustained and prolonged contact allowing peer supporters to target discussions over the support period, and choose when it was most appropriate to discuss a particular issue:

*‘You'd move on to feeding out and about because you're part of that with them, they'd be going out and experiencing that and then you'd be part of, you know, growth spurts as you'd see them at that time' *(Peer Supporters).

In turn, the peer supporter's insider knowledge of their families and lives, and *'friendship' *led to women feeling *'cared about', 'comfortable' *and *'easy' *to raise issues with peer supporters. Star Buddies also highlighted how these women were more likely to perform more intimate tasks in front of them, e.g. expressing milk. Furthermore, whilst Star Buddies have always encouraged women to maintain contact after the period of support, these instances were far more prevalent amongst the women who received incentives:

*‘...And I've found the women I've discharged, a lot of them are still getting in touch with me*.

*....There's loads of that, it's never happened before, yes*.

*....You know, further questions throughout the journey, are coming back to me' *(Peer Supporters).

#### Encouraging sensitive dialogues and opportunities for support

As the incentives created opportunities to meet up when no specific concerns were identified, the discussions often delved into a whole host of wider breastfeeding and non-breastfeeding issues. More emotive topics were raised such as bed-sharing, smoking, alcohol consumption, acquiring a tattoo and managing breastfeeding during formal occasions. Women and peer supporters referred to how the repeated contacts enabled trust to be forged within their relationships. Moreover, the trust in their peer supporters led women to seek out their opinion on personal or family issues (e.g. relationship issues, mental health concerns); maintain contact when on holiday and *'open up' *more than within their personal networks:

*‘He *(husband) *was getting a bit frustrated, so I couldn't really vent as much to him. So as soon as X *(Star Buddy) *came round I was like, just let rip. So yes, I definitely did look forward to it' *(Lucille).

These dialogues encouraged tailored support to be provided; together with referrals into appropriate services:

*‘I was supporting a muslin lady who felt really isolated but I don't think she'd have ever told me that if she hadn't have built up a relationship. There was sort of racial abuse every time she set out of her door. So we got an ethnic inclusion worker and they're supporting her with a house move ......but to me that was way more than breastfeeding. I don't think she would have trusted me if I hadn't been seeing her so regular' *(Peer Supporter).

Finally, home contacts enabled peer supporters to regularly access women's personal networks including partners and family members providing opportunities to harness their support and encouragement:

‘*Then you can spend time telling the dads ways to support the mums and showing them what to look for' *(Peer Supporters).

#### Being rewarded

Overall, the majority of women reported how the gifts per se did not alter their decision or intention to breastfeed:

*‘It's *(gifts) *been really, really nice but breastfeeding is so important to me that I can't imagine stopping .....I already know that I'm breastfeeding for a year minimum and that's it. So .....I wasn't going to be persuaded by gifts but they were very lovely all the same and I'm very grateful' *(Sandy).

These gifts did however provide intangible incentives through the pleasure they provided. Women considered the gifts as to be an *'instant encouragement'*, a *'treat'*, a '*bonus' *and something to '*look forward to'*:

*‘It was fantastic, it was such a treat to get something. I mean I was just so happy to be getting her time and her advice, the fact that I was getting like a magazine and so many little treats to go along with it, was just a massive bonus really' *(Rose).

Fundamentally, the actual support the women received via the peer supporters was considered to be crucial to their breastfeeding success; '*I definitely would have given up without their support'*. Almost all of the women perceived that on-going support from the programme had enabled them to breastfed for longer. However, as breastfeeding was often experienced as arduous and difficult, on-going receipt of the gifts re-enforced and recognised their breastfeeding achievements:

*‘When you're doing something that's painful and hard work and exhausting and pins you to a sofa for hours and hours of a day and means that you're the only one who can get up and feed in the middle of the night, then I suppose it's nice to get something that's thanking you almost, telling you you're doing a good job and that you deserve to be treated' *(Lucia).

Peer supporters identified how incentives had provided them with personal and professional rewards. The in-depth nature of the relationships forged between the supporters and women led to in-depth insider knowledge of women's lives and to gain a more authentic consideration of new motherhood:

*‘I think we only generally see women the first couple of weeks, husbands are at home, everything's still euphoric, baby's brilliant....men go back to work and then women tend to find they're struggling....and generally they're the times they drop off and you wouldn't necessarily be able to contact them. Whereas now because you're in, you can see it, and I was really shocked by the amount of women, by about week four or five were sort of hitting rock bottom' *(Peer Supporters).

Peer supporters were also more frequently challenged by the new and/or unfamiliar breastfeeding issues being raised, and this developed their breastfeeding knowledge and skills:

*‘We can't get through the door for many reasons, so I was feeling a bit jaded that actually my skills......sat at a phone just going through how many times your baby's weed and pooed and is everything going OK.....whereas this, it's sort of put a bit of blood in our.... it gave us a bit..... oh I'm using loads of my skills now' *(Peer Supporters).

Whilst the development of professional-based and person-centred capacities had been an unexpected feature of the incentives, the Star Buddies greatly appreciated the *'buzz' *it had created. The incentive intervention was considered to have harnessed the peer supporters enthusiasm and motivation for their role; enabling them to be the supporters they had envisioned:

*‘It's just doing what we're meant to do and what we're trained to do in a really valuable, meaningful way. Face to face makes all the difference, that's what it is' *(Peer Supporters).

## Discussion

The findings of this study suggest that the nature and quality of the gifts facilitated regular face-to-face contact between the peer supporters and women, increasing connections and social opportunities within and between the women, families, peer supporters and health professionals. Meaningful relationships were formed between peer supporters and mothers as they were *'on the journey together'*. These relationships encouraged dialogues around sensitive issues, enabling targeted and authentic support to be provided in this disadvantaged community, with rewards for both women and the peer supporters. This is supported by the programme's descriptive data, which show an increase in contact time and the number of home visits during the incentive intervention. The personal and professional rewards they experienced were considered an unexpected benefit of the incentive intervention, and appear to reflect the well-established training and on-going support operating within this peer support programme.

As far as the authors are aware, this is the first qualitative data reported that explores how incentives might influence infant feeding decisions. The incentive intervention embedded within an established peer support programme is an innovative approach to try to increase breastfeeding rates in a disadvantaged area and meet government indicators for progress towards breastfeeding duration rates. The peer supporters valued and welcomed the gift giving occasions. The intervention design involved service users and the peer supporters delivering the intervention, as did the evaluation where findings were discussed and shared with participants. There are, however, several limitations to this study. Whilst attempts were made to recruit participants with varying attitudes at different points and levels of engagement with the intervention; all those who agreed to take part had fully completed or were successfully engaging with the incentive programme. This either represents a bias in how peer supporters recruited women to participate in interviews or alternatively only women with more positive experiences or more motivated to continue breastfeeding volunteered. In future studies it will be important to develop different strategies to recruit women who choose not to participate in incentive schemes. The majority of women were of White-British origin, and whilst this is representative of the ethnic make-up of the local population, this limits the transferability of the findings. Previous research has identified that the social and cultural context plays an important influence on an individual's motivation [4,28] and future research should therefore recruit women from different ethnic and socio-economic groups. Unfortunately indices of deprivation were not routinely collected for participants in the peer support programme and future research should investigate how the uptake of incentives, attrition rates and outcomes vary across different socio-economic groups. Furthermore, whilst it is possible that GTs previous evaluation of the peer support programme may have influenced the final interpretations [24], care was taken to incorporate trustworthiness through discussion with the evaluation team and the programme providers and a summary of the themes shared and validated with the participants.

The commissioning brief was for qualitative evaluation only, whereas ideally it would have included prospectively designed rigorous outcome and process evaluation to assess the intervention feasibility. As the quality of the routinely collected data accessed retrospectively is uncertain, the outcome and process data were analysed descriptively after the qualitative analysis was completed to minimise retrospective bias. Some missing data were encountered and where this was extensive, for example 20% of data on parity were missing, they were not reported. These data should be treated with caution; however they do triangulate the qualitative findings and suggest that the incentive intervention did increase home visit and contact time with peer supporters. No conclusions can be drawn from the impact upon breastfeeding intentions, or outcomes and further research including an assessment of cost-effectiveness is needed.

The fact that the incentive intervention slightly increased partial participation rather than full completion of the full programme of the peer support supports findings from the WIC studies [14,15,17,19]. Overall, however, the findings identified that it was not the gifts per se that motivated these women to breastfeed. As these women were/had fully engaged with the intervention, they may well have already been highly motivated to breastfeed. However, as these extrinsic rewards provided recognition of their breastfeeding achievements, were satisfying and meaningful, and peer supporters provided empathic, individualised care and support, women's motivation to breastfeed may have been enhanced. The descriptive data supports this, as there did not appear to be any change in the proportion of women breastfeeding at 6-8 weeks across the two groups, although a slightly higher proportion of women who received the incentives were reported to be breastfeeding exclusively (75.5% versus 68.5%). This fits with evidence that lay support has a greater effect on the exclusivity of breastfeeding than initiation or duration [29,30]. Furthermore, the routinely collected 6-8 breastfeeding duration data demonstrates improvement; with the highest breastfeeding duration figures at 6-8 weeks being reported over the incentive intervention period (29.9%). Whilst the incentive intervention enabled increased contact between the peer supporters and the mothers, it is very difficult to elicit the actual benefits of the incentive and/or the benefits of increased contact. As the study only recruited women who had/were engaging with the incentive intervention, no conclusions can be drawn about how the gifts may or may not have motivated women to either participate in the peer support programme and/or to breastfeed. Future studies need to differentiate feeding outcomes and participant perspectives for a) incentives alone b) incentives with peer support and c) peer support alone, as well as elicit views from women who choose not to engage, or withdraw from, an incentive intervention.

Ryan & Deci's (2000) Self-Determination theory [4] proposes there are three innate needs which influence self-motivation and personality integration, namely 'competence' (belief in our capabilities to succeed), 'autonomy' (belief that outcomes are dependent on our own capabilities and volition) and 'relatedness' (connections/relationships to members of our social network)[4]. Whilst these constructs are considered to fuel intrinsic motivation, these authors propose that external influences can equally promote wellbeing and growth [4]. The constructs of autonomy and competence were evident within the Star Buddy service in terms of how the peer supporters supported and empowered women. Whilst these insights have been reported in a previous publication [24], the issue of relatedness was a key theme identified within this study. The connected relationships, enabled and enhanced via tangible (gifts) and intangible (breastfeeding support) incentives meant that: a) women were likely to trust the support provided, encouraging on-going access; b) women were likely to disclose wider socio-emotional issues and barriers that may impact upon breastfeeding c) incentives provided opportunities for peer supporters to provide tailored support, d) the reassurance, praise and feeling '*cared for' *enhanced maternal wellbeing and e) peer supporters developed professional skills and motivation within their role. These findings concur with the Darzi report [31] that incentives can recognise, reward and improve quality of service and with Johnston & Sniehotta [14] in that inexpensive gifts can operate as a social reward, and if incorporating intrinsic motivation, a self-reward.

Within the breastfeeding as well as the incentive literature it is considered that multifaceted interventions, that span pregnancy and after birth, and that utilize a variety of methods and support are more effective than a singular approach [2,30,32]. Furthermore, it is important to recognize that the perceptions, attitudes and utility of incentives will be varied across different socio-cultural groups. Indeed, in *Birth by Design *the authors argue how maternity care systems need to be studied within the cultural, historical, and societal settings in which they operate [33]. Although these findings offer invaluable insights into how an incentive intervention is received and internalized, further research to explore the underlying motivations and intentions of breastfeeding women is essential [28]. Questions remain about the effectiveness and cost-effectiveness of gifts or incentives to increase breastfeeding duration: are they more effective if targeted to certain socio-economic or ethnic groups as well as whether incentives facilitate intrinsic motivation as well as provide extrinsic motivation?

## Conclusion

This study suggests that gifts may be unlikely to incentivize women to initiate or sustain breastfeeding for longer, but might improve women's overall wellbeing. In addition, incentives facilitate home access for peer supporters to develop health enhancing relationships where needs can be assessed and support provided, with onward referral to other agencies when indicated. Delivering gifts can enable peer supporters to fulfill their potential, within an established peer support model providing training, supervision and mentoring. These findings have relevance for health promotion and disease prevention practice and policy, where peer and community networks can assist in achieving healthy lives and healthy people, particularly in more disadvantaged areas [34].

## Competing interests

There are no financial or non-financial competing interests (political, personal, religious, ideological, academic, intellectual, commercial or any other) to declare in relation to this manuscript.

## Authors' contributions

GT was involved in design, data collection, analysis, reporting of the data and lead author on the manuscript. FD was involved in drafting and critical review of the manuscript. MH was involved in preparation and analysis of the descriptive data. PH made significant contributions to the design and conception of the manuscript and analysis of descriptive data.

## Authors' information

GT is currently working as a Research Fellow in the Maternal and Infant Nutrition and Nurture Unit (MAINN) at UCLan and was the project lead in undertaking an in-depth evaluation of the Star Buddies breastfeeding peer support project and incentive intervention project (undertaken over 2009 to 2011). PH is a Senior Clinical Research Fellow in the Health Services Research Unit at the University of Aberdeen and a part time General Practitioner. The Health Services Research Unit is supported by the Chief Scientist Office (CSO) of the Scottish Government Health Directorates. FD is Professor of Maternal and Infant Health and Director of MAINN. MAH is a Senior Lecturer in Medical Statistics and is a chartered statistician of the Royal Statistical Society with maintained professional certification.

## Pre-publication history

The pre-publication history for this paper can be accessed here:

http://www.biomedcentral.com/1471-2393/12/22/prepub
